# cDCD organ donation pathway of Romagna Local Health Authority: strategic planning, organizational management, and results

**DOI:** 10.1007/s44250-023-00022-0

**Published:** 2023-03-30

**Authors:** Alessandro Circelli, Marta Velia Antonini, Andrea Nanni, Manila Prugnoli, Emiliano Gamberini, Stefano Maitan, Claudio Gecele, Lorenzo Viola, Luca Bissoni, Giovanni Scognamiglio, Luca Mezzatesta, Carlo Bergamini, Luca Gobbi, Manlio Cosimo Claudio Meca, Gabriela Sangiorgi, Marcello Bisulli, Martina Spiga, Veruska Pransani, Daria Liuzzi, Valentina Fantini, Fausto Catena, Emanuele Russo, Vanni Agnoletti

**Affiliations:** 1 Anesthesia and Intensive Care Unit, Bufalini Hospital-AUSL della Romagna, Cesena, Italy; 2 Department of Biomedical, Metabolic and Neural Sciences, University of Modena & Reggio Emilia, Emilia-Romagna, Italy; 3 Transplant Procurement Management-AUSL della Romagna, Cesena, Italy; 4 Anesthesia and Intensive Care Unit, Infermi Hospital, AUSL della Romagna, Rimini, Italy; 5 Intensive Care Unit, Morgagni-Pierantoni Hospital-AUSL della Romagna, Forlì, Italy; 6 Anesthesia and Intensive Care Unit, Santa Maria delle Croci Hospital, Ravenna, Italy; 7 Emilia-Romagna Regional Transplant Center Coordination, Bologna, Italy; 8 Interventional Radiology Department, Bufalini Hospital-AUSL della Romagna, Cesena, Italy; 9 General and Emergency Surgery, Bufalini Hospital-AUSL della Romagna, Cesena, Italy

## Abstract

The introduction of pathways to enrol deceased donors after cardio-circulatory confirmation of death (donation after circulatory death, DCD) is expanding in many countries to face the shortage of organs for transplantation. The implementation of normothermic regional reperfusion (NRP) with warm oxygenated blood is a strategy to manage in-situ the organs of DCD donors. This approach, an alternative to in-situ cold preservation, and followed by prompt retrieval and cold static storage and/or ex-vivo machine perfusion (EVMP), could be limited to abdominal organs (A-NRP) or extended to the thorax (thoraco-abdominal, TA-NRP. NRP is also referred to as extracorporeal interval support for organ retrieval (EISOR). The use of EISOR is increasing in Europe, even if variably regulated. A-NRP has been demonstrated to be effective in decreasing the risk associated with transplantation of abdominal organs from DCD donors, and was recommended by the European Society for Organ Transplantation (ESOT) in a recent consensus document. We aim to explain how we select the candidates for DCD, to describe our regionalized model for implementing EISOR provision, and to introduce the health care professionals involved in this complex process, with their strictly defined roles, responsibilities, and boundaries. Finally, we report the results of our program, recruiting cDCD donors over a large network of hospitals, all pertaining to a Local Health Authority (Azienda Unità Sanitaria Locale, AUSL) in Romagna, Italy.

## Introduction

The introduction of pathways to enrol deceased donors after cardio-circulatory confirmation of death (donation after circulatory death, DCD) [1–4], which is still not allowed globally, is increasing in many countries in answer to the shortage of organs for transplantation.

The implementation of normothermic regional reperfusion (NRP) with warm oxygenated blood is a strategy to manage in-situ the organs of DCD donors [5, 6]. This approach, alternative to in-situ cold preservation followed by prompt retrieval and cold static storage and/or ex-vivo reperfusion (EVP) could be limited to abdominal organs (A-NRP) or extended to the thorax (thoraco-abdominal, TA-NRP [3]. NRP is also referred to as extracorporeal interval support for organ retrieval (EISOR) [7]; the strategy include both the use of a peripheral femoro-femoral veno-arterial configuration of extracorporeal support, and aortic occlusion to prevent cerebral reperfusion [3, 8, 9]. To this end, an endovascular aortic occluder (a balloon catheter) or direct aortic cross clamping could be used [3, 8, 9]. We recently described our technique in detail [9].

The use of EISOR is increasing in Europe, even if variably regulated [10, 11]. A-NRP has been demonstrated to be effective in decreasing the risk associated with transplantation of abdominal organs from DCD donors [12–19], and was recommended by the European Society for Organ Transplantation (ESOT) in a recent consensus document [3].

We aim to describe how we select the candidates for DCD, to introduce the health care professionals involved in this complex process, with their strictly defined roles, responsibilities, and boundaries, and report on the results of our regionalized program for recruiting cDCD donors in a subregional network of hospitals, all pertaining to a Local Health Authority (Azienda Unità Sanitaria Locale, AUSL) in Romagna, Italy.

## Who is the candidate for DCD? Defining the donor and grading the associated risk

A patient is identified as “possible” deceased organ donor [11], if presenting with a devastating brain injury or lesion, or with circulatory failure refractory to maximal treatment (conventional or extracorporeal cardiopulmonary resuscitation as indicated on a case-by-case base) [11, 21], and if potentially suitable to be evaluated for organ donation. The pathway for DCD organ donation is embarked on in any of the ICU of the hospitals referring to the EISOR center when an adult patient presents with devastating brain injury or lesion, but with criteria for neurological determination of death (donation after neurological, DND) not met and unlikely to evolve to DND, or patients presenting with circulatory failure refractory to maximal treatment (conventional or extracorporeal cardiopulmonary resuscitation as indicated on a case-by-case base) [11, 21].

Conventionally, DCD donors are categorized according to the modified Maastricht classification [22, 23]. The main categorization refers to a description of DCD donors as controlled or uncontrolled. Despite controlled DCD programs being more challenging from an ethical and legal perspective, these allow planning and coordination of the efforts focused on donor evaluation, and subsequent eventual organ procurement [24]. Moreover, in controlled DCD (cDCD) donors, warm ischemia time could be strictly monitored, and strategies for in-situ regional perfusion can be implemented. All of these could contribute to improved outcomes of grafts procured and transplanted from cDCD.

**Fig. 1:**
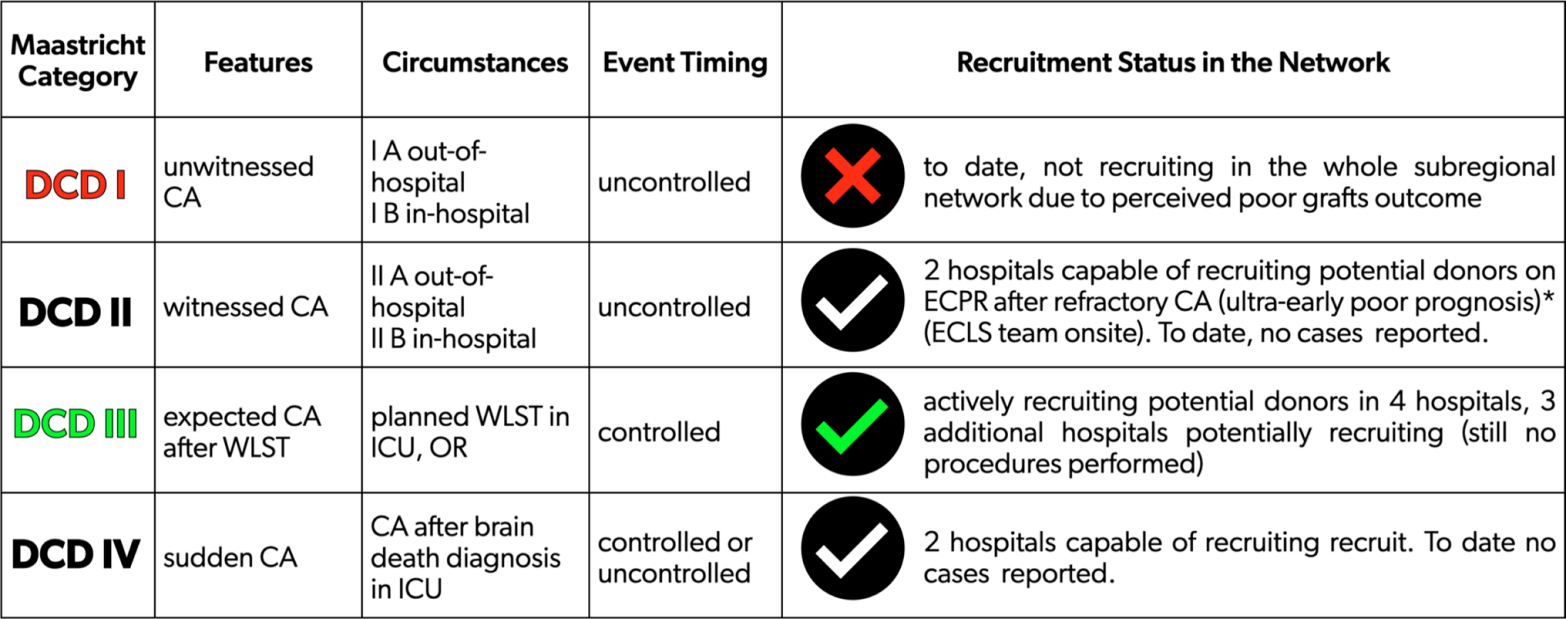
Modified Maastricht Classification for DCD donors (Kootstra et al., Thuong et al.), and potential for enrolment in the network of hospitals pertaining to the Local Health Authority of the Romagna Area. *DCD* donation after cardio-circulatory determination of death, *CA* cardiac arrest, *ECPR* extracorporeal cardiopulmonary resuscitation, *ECLS* extracorporeal life support, *WLST* withdrawal of life sustaining treatment, *ICU* intensive care unit, *OR* operating room

To date, our program is only actively enrolling Maastricht category III cDCD donors, despite being potentially able to recruit other categories (Fig. 1).

In potential cDCD, if further active treatment is perceived as no longer in the best interests of the patient and not consistent with patients’ wishes and preferences [25], WLST is considered, in full agreement with scientific societies’ recommendations and with national guidelines. The ICU team caring for the patient approach the local Hospital Procurement Team, including specialized physicians and nurses, who report the case to the Regional Transplant Reference Center (Centro di Riferimento Trapianti Emilia Romagna, CRT-ER), in order to assess the suitability of the potential donor candidate.

A thorough clinical evaluation is initiated, in order to exclude the actual risk of donor-to-recipient disease transmission, and to understand the patient’s expressed wishes; in uncontrolled donors this complex and time-consuming evaluation only occurs after a determination of death. The assessment is performed under the guidance of the Regional Transplant Reference Center, providing a basic consultation and, eventually, second opinions to define donor suitability and stratify any risk associated with transplantation. Moreover, the CRT-ER access the national database collecting consent/refusal statements toward organ donation, the Informative Transplant System (Sistema Informativo Trapianti, SIT).

If no wishes were previously expressed the family is approached by the local Hospital Procurement Team, and they are allowed to express their opposition to organ and/or tissue donation.

According to the Italian National Transplant Centre (Centro Nazionale Trapianti, CNT) guidelines, the patient is considered a-priori unsuitable if he/she opted out and/or if they present with any among selected malignant neoplastic disorders, hematologic diseases, or infective conditions that absolutely disqualify the donor due to an unacceptable risk to any recipient [26].

If no impediments are detected, an initial process of matching donor-potential recipients is undertaken by CRT, as the evaluation process proceeds and WLST is planned.

After determination of death according to cardio-circulatory criteria, if no opposition or clinical ineligibility have emerged, and the total warm ischemia time is suitable to allow for organ transplantation, the donor is considered “eligible”. Cannulation is performed and, after aortic balloon inflation, EISOR is initiated. The detailed technique was previously described [10]. The donor is considered “actual” as skin is incised to initiate surgical intervention with the intention of procuring the organ(s), or if at least one organ is effectively procured.

DCD associated risk could be standard or non-standard; non-standard risk is furtherly classified as negligible, or acceptable. Risk is negligible as long as no known conditions affecting transplant outcomes or requiring treatment are identified. No restrictions apply to solid organ transplantation, but the recipient needs to sign an informed consent. Risk is deemed acceptable if the presence of a clinical condition that could affect outcome, if left untreated, is identified. Potential recipients should be properly matched; restrictions apply to solid organ transplantation, but recipients need to sign a form giving informed consent. For acceptable risk candidates, tissue donation is not allowed. If the risk is still deemed acceptable, but high, recipients are selected among high-risk profile patients with referral to disease severity and/or urgency, as the risk/benefit ratio favours the second.

The CNT criteria aimed to proceed with a high level of caution, and are slightly more restrictive compared with the European selection criteria [26, 27].

During the whole procedure, from EISOR initiation to ex-vivo graft perfusion, the level of risk initially assigned to the donor may change according to ongoing findings and assessment of organ function, eventually preventing graft transplantation. If at least one organ is effectively transplanted, the donor is finally defined as “utilized."

**Fig. 2:**
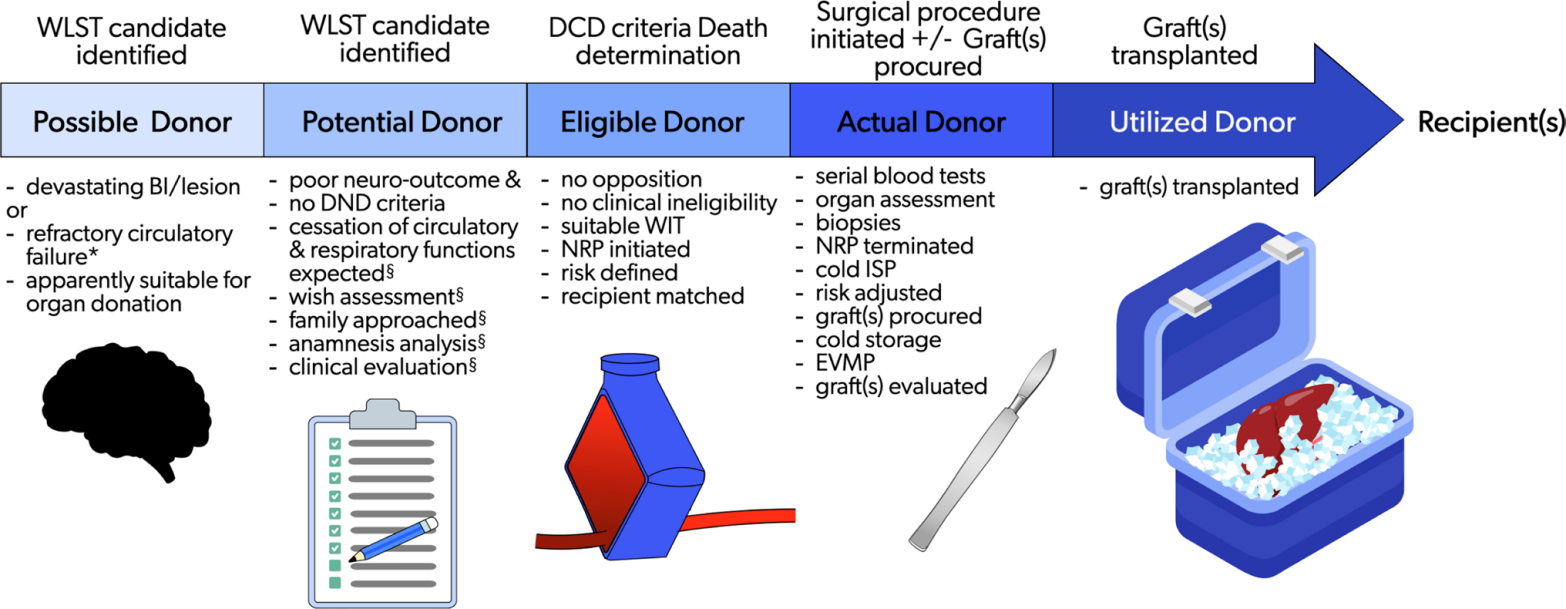
Identification of cDCD donor and evolving definitions during assessment for suitability. Only a fraction of the donors move to the subsequent phase, as the pathway might be interrupted at any stage due to emerging awareness of clinical findings or opposition to organ donation. *cDCD* controlled donation after cardio-circulatory determination of death, *WLST* withdrawal of life sustaining treatment, *BI* brain injury, *DND* donation after neurological determination of death, *WIT* warm ischemia time, *NRP* normothermic regional perfusion, *ISP* in situ perfusion, *EVMP* ex-vivo machine perfusion, referral center; *TX* transplantation

These evolving definitions, consistent with the currently recommended European definitions [3, 20], are depicted in the Fig. 2. According to the CNT definitions, the eligible donor is furtherly defined “reported” as after determination of death the case is officially included in the CRT Registry, and “procured,” if no opposition or clinical ineligibility have emerged [28, 29].

## Who is behind organ procurement from DCD donors? Multidisciplinary teams, roles, responsibilities, and boundaries

Despite the obvious extensive collaboration to facilitate organ donation and procurement, to avoid any ethical conflict of interest, the responsibility for patient management leading to WLST, and WLST itself, the donor evaluation, leading to eventual organs allocation, and the EISOR procedure are managed by different teams. These are, respectively:

- the ICU team
- the procurement team, involving the hospital team, the local health team, and the regional reference center
- the extracorporeal life support (ECLS) team

**Fig. 3:**
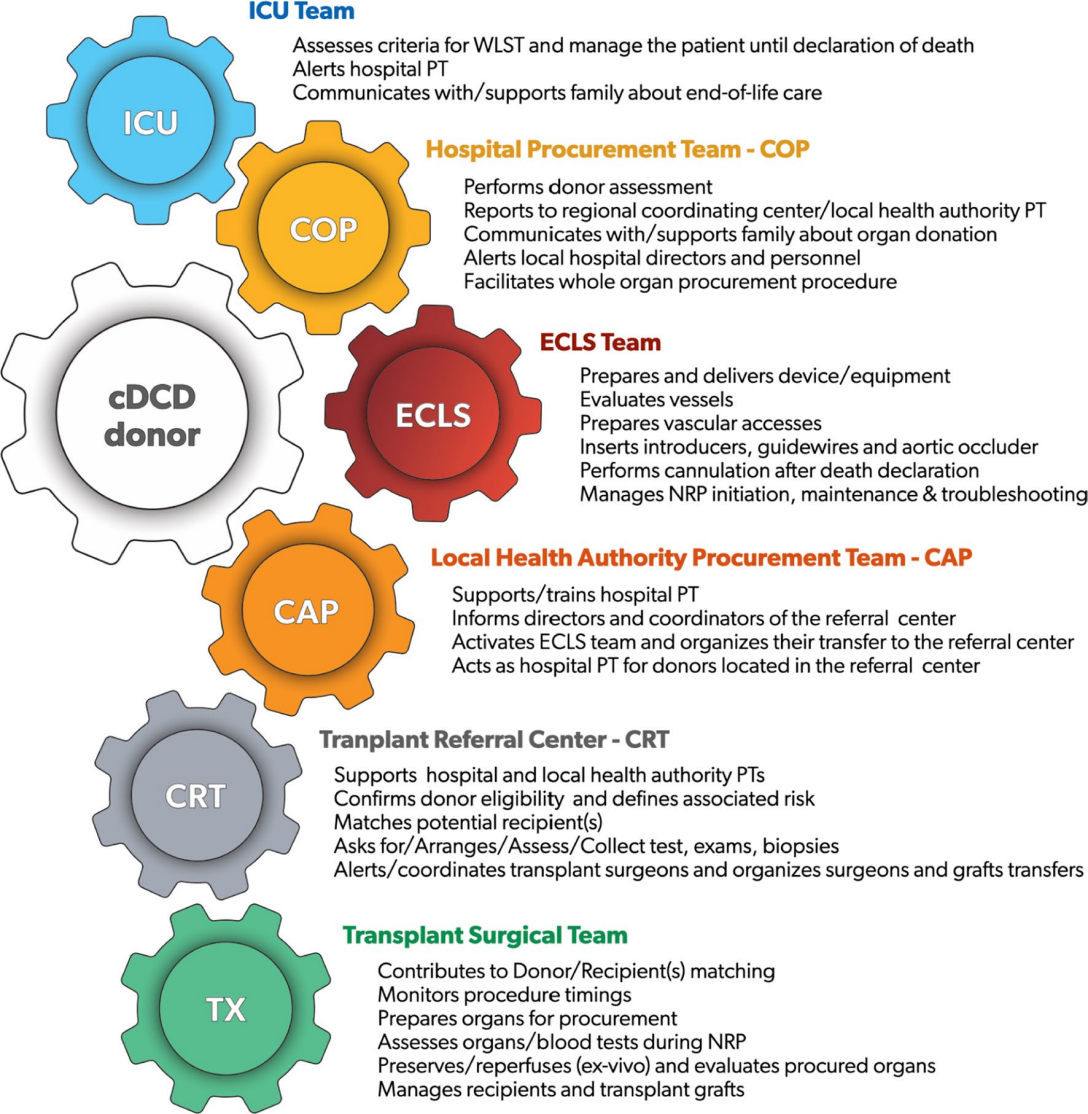
Simplified graphic of major teams involved in cDCD organ donation pathway and their main tasks; random order. *cDCD* controlled donation after cardio-circulatory determination of death, *ICU* intensive care unit, *WLST* withdrawal of life sustaining treatment, *PT* procurement team, *COP* local hospital procurement team, *CAP* local health authority procurement team, *ECLS* extracorporeal life support, *NRP* normothermic regional perfusion, *CRT* regional transplant referral center, *TX* transplantation

A summary of main tasks and responsibilities of major teams involved in DCD organ procurement is included in Fig. 3.

### The ICU team

The ICU team involves intensivists, critical care nurses, and other allied health care professionals caring for the critical care patient considered for WLST and eventually for subsequent organ donation. When clinical criteria are met [11, 20, 21] the multidisciplinary team discusses the possible futility of ongoing or additional therapies and procedures, eventually coming to an agreement that the patient would benefit most from a WLST.

Before WLST, the responsible physician alerts the hospital procurement team.

The ICU team strictly monitors and manages the possible, then potential, donor to prevent, detect and treat any discomfort and pain, while optimizing end-organ perfusion. Palliative care to avoid any suffering persists during WLST. The intensivist in charge interrupts any life support, whether pharmacological or mechanical, when the preparation phase for post-mortem implementation of EISOR ends [9], then verifies and certifies death according to DCD criteria. [30–32]

The ICU team communicates with the family about futility and end-of life care, investigating the patient’s preferences, if known, to be integrated in the decision-making process. Family needs are supported, and their presence at the bedside favored until asystolia occurs, if they wish.

### Hospital procurement team

The hospital procurement coordination team (coordinamento ospedaliero per il procurement, COP), includes a head intensivist, a procurement coordinator nurse, and a group of transplant procurement management (TPM) experienced nurses [30, 32, 33]. COP interfaces continuously with the Transplant Reference Center and Local Health Authority Procurement Team. The main tasks of the COP are taking care of the donor during the whole pathway, reporting the case, and performing a thorough assessment under CRT guidance, supervising WLST and determination of death to ensure that ischemia times are suitable for subsequent organ procurement. COP performs full donor assessment (anamnesis, physical examination, radiological assessment, lab tests), involving essential consultants as needed, to confirm eligibility.

The hospital procurement coordination team informs the medical director and nursing and healthcare professions director of the hospital, to organize pre-procedural arrangements, and alerts/organizes hospital physicians, nurses, other HCPs and support personnel required to accomplish the procedure. Moreover, COP communicates with the family about organ donation, supporting their needs.

During EISOR and surgical intervention, requests for additional examinations and results delivery, from lab tests to biopsies, to further define risk definition.

### Local Health Authority procurement team

A Local Health Authority procurement coordination team (coordinamento aziendale procurement CAP) is available to start and support an organ donation program for both DND and DCD donors in all the seven hospitals of the Local Health Authority where at least one intensive care unit is available. The CAP supports COP for any eventual needs, from donor assessment and management to facilitation of EISOR procedure, also providing team member training. For newly established DCD programs, the CAP backs up COP until a local team is completely autonomous.

When the CAP is notified about a possible DCD donor in one among the seven hospital ICUs, the case is brought to the attention of both the medical and the nursing and HCPs directors of the EISOR Hub, and of the medical director and nursing chief of the EISOR hub hospital ICU, in order to authorize CAP itself and ECLS team involvement. After arrangements according to the donor characteristics and team availability, the CAP organizes team transfer and delivery of any equipment required to the ICU involved through the Local Health Authority Emergency Medical Service (EMS). A van and a driver are arranged for the round-trip (during the procedure, he is available for other delivery tasks).

**Fig. 4:**
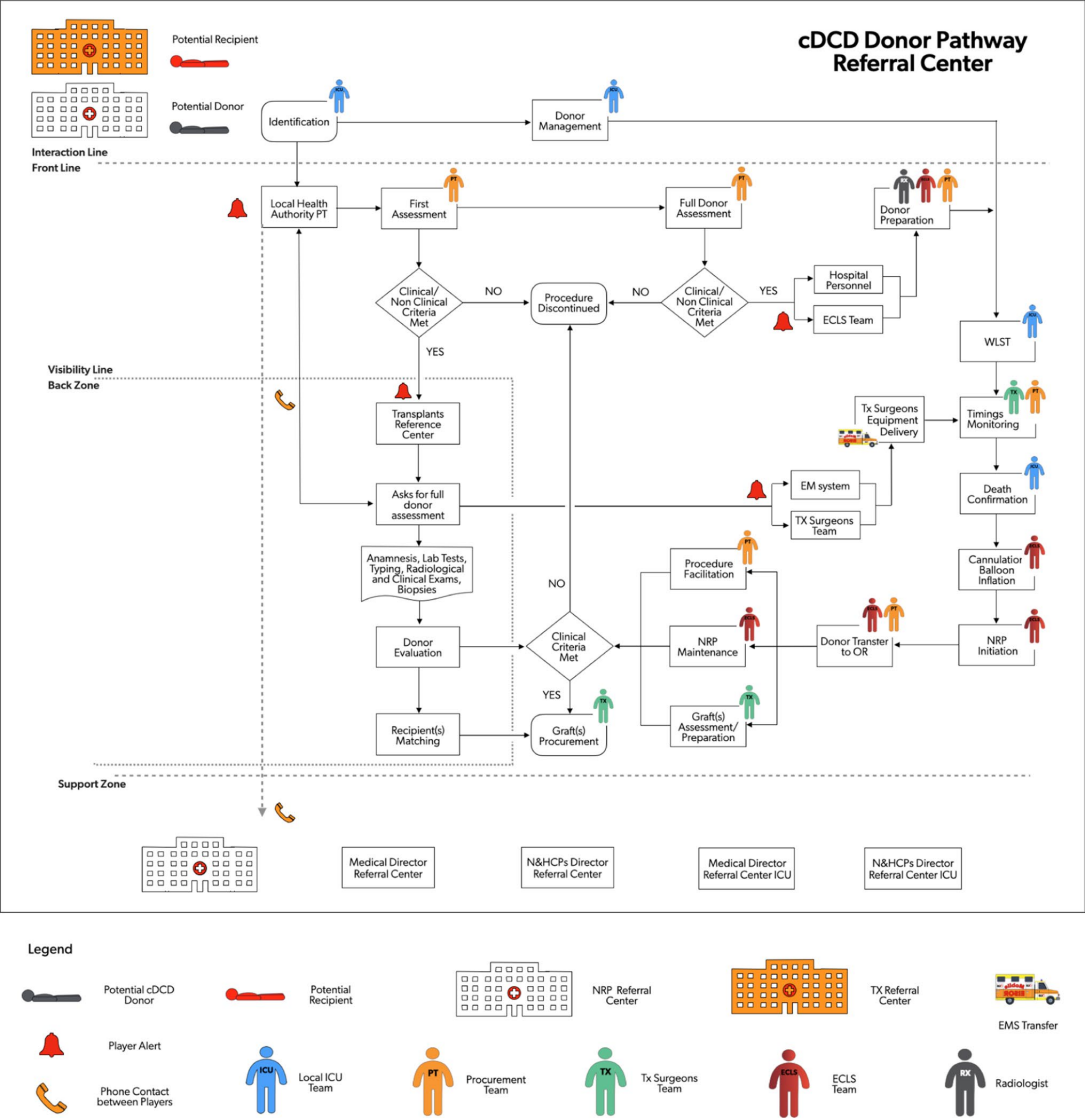
Service mapping of cDCD donor pathway for procedures occurring in the referral center. *cDCD* controlled donation after cardio-circulatory determination of death, *PT* procurement team; *ECLS* extracorporeal life support, *WLST* withdrawal of life sustaining treatment, *TX* transplantation, *EMS* emergency medical system, *NRP* normothermic regional perfusion, *OR* operating room, *N&HCPs* nursing and allied healthcare professionals, *ICU* intensive care unit

**Fig. 5:**
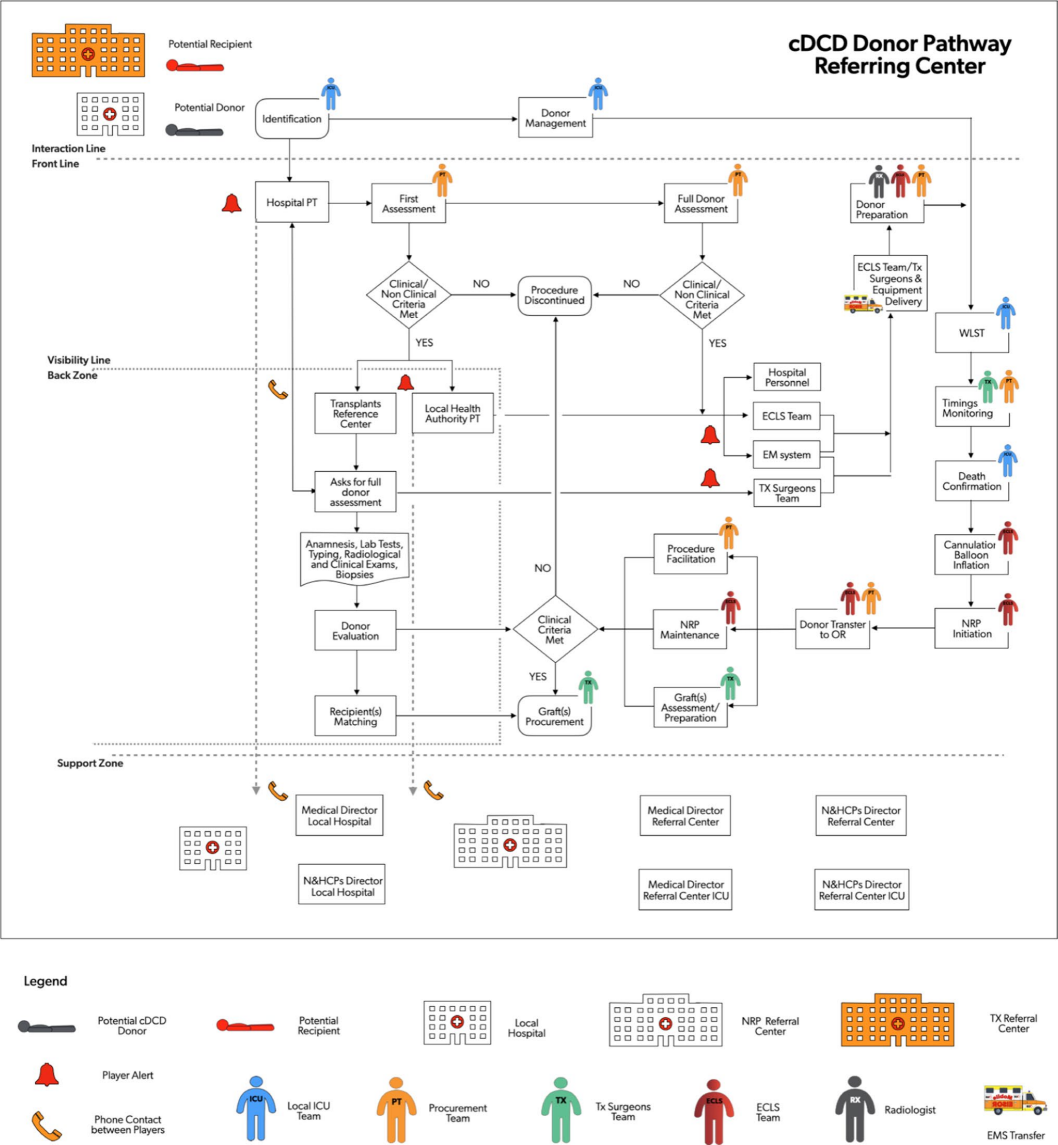
Service mapping of cDCD donor pathway for procedures occurring in the referring center. *cDCD* controlled donation after cardio-circulatory determination of death, *PT* procurement team; *ECLS* extracorporeal life support, *WLST* withdrawal of life sustaining treatment, *TX* transplantation, *EMS* emergency medical system, *NRP* normothermic regional perfusion, *OR* operating room, *N&HCPs* nursing and allied healthcare professionals, *ICU* intensive care unit

For DCD donors identified in the EISOR hub, the CAP and COP team members partially coincide, so the procedure is simplified (Figs. 4, 5).

### Transplant Reference Center

The Emilia-Romagna Transplant Reference Center (Centro Riferimento Trapianti dell’Emilia Romagna, CRT-ER) provides leadership and supports procurement teams, checking the SIT, and evaluating all the available data, information, reports to confirm or deny a donor’s eligibility, and to grade associated risk. If required, CRT asks POC to perform additional examinations and consultations. If donors are deemed eligible, CRT initiates to match potential recipient(s).

CRT interfaces with the two equipes of transplant surgeons available in the Region for DCD donors to start matching potential recipient(s), and organizes their transfer to the procedure site, and back to their hospitals, eventually with the graft(s) if procurement successful. During the procedure, CRT collects blood test results, surgeons’ intraoperative assessments and findings, and arranges for liver and/or renal biopsies to be analyzed in real time. According to all available data, CRT adjusts the donor’s risk status and adapts recipient matching accordingly.

### ECLS Team

The ECLS team includes a group of intensivists, nurses and perfusionists experienced in extracorporeal support, taking care of all aspects, from device/equipment preparation and delivery to EISOR implementation and management. Our team and technique have been previously described in detail [9].

During WLST, they evaluate and prepare femoral vessels for subsequent cannulation, and insert the aortic occluder in the potential DCD donor. After declaration of death [30–32], the ECLS team cannulates the eligible donor and initiates EISOR. They maintain and monitor normothermic regional perfusion, performing seriated blood samples according to POC request, and provide troubleshooting if and as required. The extracorporeal organ support ends when, as organs are successfully isolated after thorough evaluation, in-situ cold perfusion and preservation could be initiated.

A dedicated team of experienced surgeons thoroughly evaluate the donor to define the risk, and match organs with potential recipients. During surgery, the team assesses organs both macroscopically, though palpation, and performing biopsies, and continuously re-evaluates their function and the trend of blood tests. After procurement, the surgeons prepare, preserve, and reassess grafts, before confirming their suitability for transplantation.

Moreover, a radiologist and a vascular surgeon are also pre-alerted and are available, on-request, to support respectively guidewire/balloon positioning, and surgical cannulation/management of major cannulation related complication, if difficulties are encountered during the procedure.

## Why build and implement a DCD program? The rationale is in the data!

**Fig. 6:**
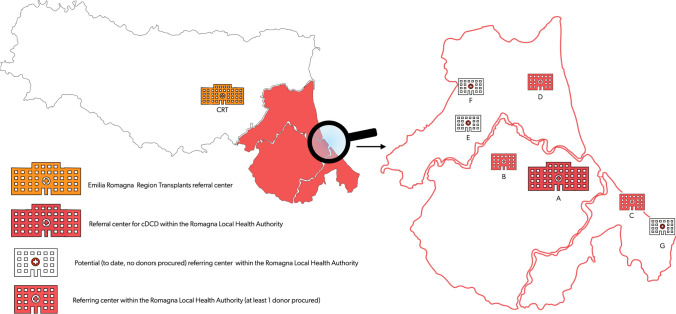
Map of the Emilia Romagna Region, Italy; Romagna area is highlighted in red. The hospitals included in the Local Health Authority Network are depicted; hospitals that have procured DCD donors, to date, are listed from A to D according to the date of first DCD donor enrollment; potential referring center that did not procure DCD donors, to date, are listed from E to G in alphabetical order. *DCD* donation after cardio-circulatory determination of death, *cDCD* controlled DCD

**Fig. 7:**
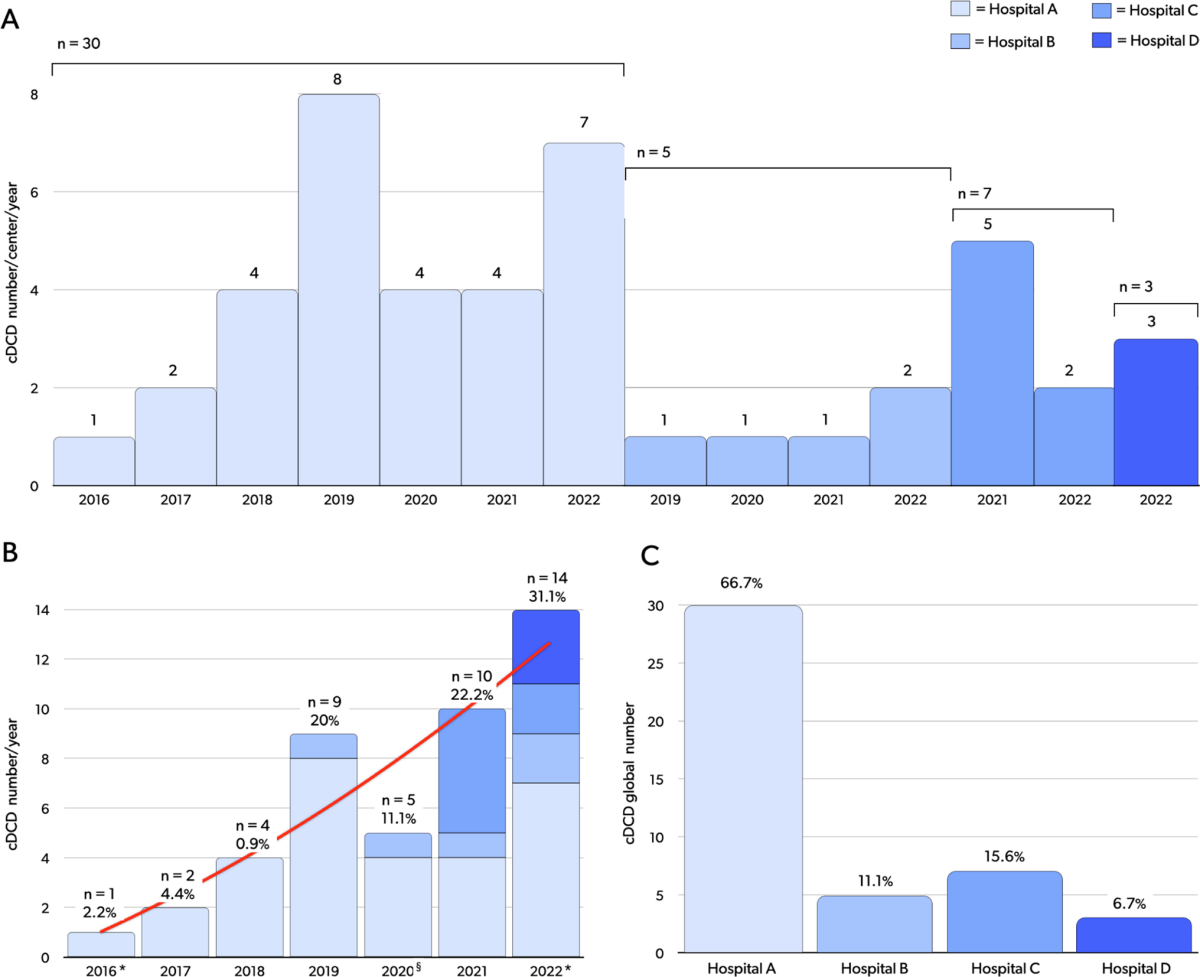
cDCD donors enrolled in the hospital pertaining to the Local Health Authority of the Romagna Area. Four out of seven hospitals procured donors, to date. **A**: cases per year/per hospital. **B**: total cases per year. **C**: total cases per hospital. *cDCD* controlled donation after circulatory death. Hospitals are listed from **A** to **D** according to the date of first DCD donor enrolment. * For 2016, data from September 1 to December 31; for 2022, data from January 1 to August 31. § For 2021, numbers could have been impacted by first waves of Coronavirus 2019 pandemic

**Fig. 8:**
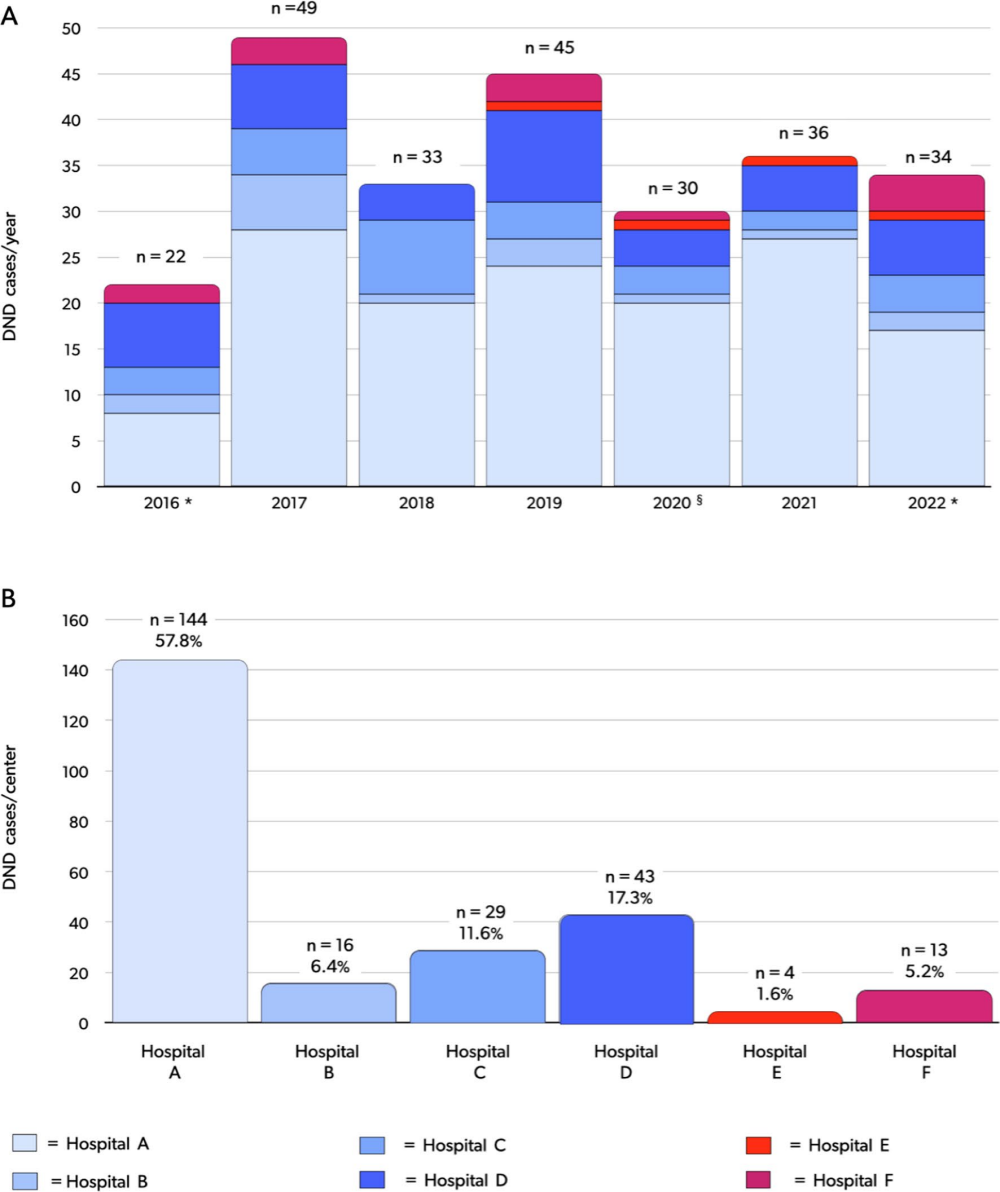
DND donors enrolled in the hospital pertaining to the Local Health Authority of the Romagna Area. Six out of seven hospital procured donors, to date. **A** total cases per year. **B** total cases per hospital. Hospitals that have procured DCD donors, to date, are listed from **A** to **D** according to the date of first DCD donor enrolment; potential referring center that did not procure DCD donors, to date, are listed from E to F in alphabetical order. *DND* controlled donation after circulatory death, *DCD* donation after cardio-circulatory determination of death. * For 2016, data from September 1 to December 31; for 2022, data from January 1 to August. § For 2021, numbers could be impacted by first waves of Coronavirus 2019 pandemic

The Romagna Local Health Authority, including a network of seven hospitals, initiated the DCD program in September 2016. The CRT-ER identified the Bufalini Hospital in Cesena as the EISOR training center for the Emilia Romagna region, and referral center for the Romagna region [34]. All the hospitals of the network are potentially able to enrol both DND and cDCD donors, the latter thanks to the support of the mobile ECLS center of the referral center. To date, six hospitals have recruited DND donors, and four hospitals have recruited cDCD donors (Figs. 6, 7, 8). From September 2016 to August 2022 we identified and reported 46 cDCD donors to the CRT-ER Registry. Of these, 45 (97.8%) became actual donors, while one eligible donor (2.2%) was rejected as their level of risk increased to unacceptable. Detailed center-by-center analysis is included in Fig. 7A–C.

43 donors (93.5%) became utilized donors: only two (4.4%) actual donors were rejected as their level of risk increased to unacceptable. These relative percentages are higher compared with the fraction of eligible DND donors becoming actual and utilized over the same period, respectively 63.4% and 56.5% (Table 1). cDCD donors have a prolonged ICU stay before WLST, and eventual clinical absolute contra-indications are often known before WLST. Moreover the dying patient wish and the eventual family opposition could be investigated [21].

**Table 1:** Ratio between eligible, actual, and utilized DND and cDCD donors recruited since the launch of the cDCD program of the AUSL della Romagna in late 2016 to August 2022

	Eligible	Actual	Utilized
cDCD n (%)	46	45 (97.8)	43 (93.5)
DND n (%)	393	249 (63.4)	222 (56.5)

*DND* donation after neurological determination of death, *cDCD* controlled donation after circulatory determination of death

The ratio between cDCD and total donors, including DCD and DND, evolved significantly over the years (Table 2): from 4.3% over total actual donors in late 2016 (total actual donors from September 2016 to December 2016) to 29.2% in 2022 (total actual donors from January 2022 to August 2022), considering the results of the whole network. The data present a high inter-center variability, mainly related to the specific characteristic of the hospitals, in terms of category of patients usually admitted (i.e. patients with traumatic brain injuries and other neurocritically ill patients), and on the availability of neurosurgery, impacting on the number of eligible DND donors.

**Table 2:** Actual DND and DCD donors recruited since the launch of the DCD program of the AUSL della Romagna in late 2016: hospitals are listed from A to B according to the year of first DCD donor enrolment (early to late)

Hospital	Year	Actual DND	Actual DCD	Total donors	DCD/Total donors ratio, %	
					Per year	Global^b^
Hospital A	2016^a^	8	1	9	11.1	16.9
	2017	28	2	30	6.7	
	2018	20	4	24	16.7	
	2019	24	8	32	25.0	
	2020^§^	20	4	24	16.7	
	2021	27	4	31	12.9	
	2022^a^	17	7	24	29.2	
Hospital B	2016^a^	2	0	2	0.0	43.75 (25)
	2017	6	0	6	0.0	
	2018	1	0	1	0.0	
	2019	3	1	4	25.0	
	2020^§^	1	1	2	50.0	
	2021	1	1	2	50.0	
	2022^a^	2	2	4	50.0	
Hospital C	2016^a^	3	0	3	0.0	47.9 (25)
	2017	5	0	5	0.0	
	2018	8	0	8	0.0	
	2019	4	0	4	0.0	
	2020^§^	3	0	3	0.0	
	2021	3	5	8	62.5	
	2022^a^	4	2	6	33.3	
Hospital D	2016^a^	7	0	7	0.0	33.3 (4.8)
	2017	7	0	7	0.0	
	2018	4	0	4	0.0	
	2019	10	0	10	0.0	
	2020^§^	4	0	4	0.0	
	2021	5	0	5	0.0	
	2022^a^	6	3	9	33.3	
Hospitals A–D	2016^a^–2022^a^	233	45	278	–	35.46 (17.9)

*DND* donation after neurological determination of death, *DCD* donation after circulatory determination of death

^a^For 2016 only data from September 1 to December 31 are included; for 2022, only data from January 1 to August 31

^b^DCD/Total donor ratio is calculated over the years of actual DCD donor enrolment for hospitals that started after 2016

^§^the numbers for 2020 are likely to be influenced by COVID-19 pandemic

This evolving ratio, and the exponential increase over the year, and the high number of utilized cDCD donors, suggest that the program is effective in increasing the availability of grafts suitable for transplantation.

## How do you build and implement a DCD program? Regionalization could be the answer!

The implementation of EISOR could improve the outcome of grafts from DCD donors [3, 4, 16, 35–39], and its use in mandatory in the settings of expected prolonged warm ischemic times [28, 29, 40].

The technique is relatively complex from an ethical, organizational, and technical point of view; moreover, it is costly, in terms of both human and material resources. Multidisciplinary, experienced, and coordinated teams of healthcare providers are pivotal to ensure a successful procedure. We propose an organizational strategy based of regionalization, to provide reperfusion in cDCD donors in local hospitals [9], avoiding the need to move the patient before the death with the unique purpose of organ transplantation, which could be ethically debatable and clinically unsafe. We demonstrated that this organization, including a referral hospital and a network of referring hospitals, sharing personnel, expertise, and equipment is feasible, effective and efficient [9, 28, 29]. In fact, we are able to enrol cDCD donors in the Local Health Authority without the need for local full ECLS teams and ECLS related equipment, sharing personnel, expertise, and equipment; the detailed analysis of associated costs is currently being performed, and will be presented in a future paper. A related program of on-the-field training, associated with regular theoretical education, is ongoing, in parallel, to increase the pool of providers able to support the procedure. This enables the provision of a partial autonomization of local teams (i.e. for procurement and cannulation), in order to avoid an excessive strain on the referral hospital team, and to enhance the participation of local providers, but always ensuring the presence of a full and fully capable team.

These two features of the program, EISOR delivery and local team involvement, outline a regional organization avoiding the limitations of a conventional hub and spoke model [41–43] in the setting of a procedure that is time-sensitive and complex, and whose outcomes could be case volume-related [44–46].

The implementation of this model requires the defeat of a personalistic, hospital focused approach that is blindly aimed at increasing the visibility of a single institution. It must find its rationale in its effectiveness in increasing the pool of successful donors.

The further extension of the program to other hospitals in the network that still did not refer cases, and the eventual inclusion in the program of uncontrolled donors, could be future goals to be pursued.

## Data Availability

Not applicable.
